# Oncolysis by paramyxoviruses: multiple mechanisms contribute to therapeutic efficiency

**DOI:** 10.1038/mto.2015.11

**Published:** 2015-07-22

**Authors:** Olga V Matveeva, Zong S Guo, Svetlana A Shabalina, Peter M Chumakov

**Affiliations:** 1 Biopolymer Design LLC, Acton, Massachusetts, USA; 2 Engelhardt Institute of Molecular Biology, Moscow, Russia; 3 Division of Surgical Oncology, University of Pittsburgh Cancer Institute, Pittsburgh, Pennsylvania, USA; 4 National Center for Biotechnology Information, National Library of Medicine, National Institutes of Health, Bethesda, Maryland, USA; 5 Lerner Research Institute, Cleveland Clinic Foundation, Cleveland, Ohio, USA

## Abstract

Oncolytic paramyxoviruses include some strains of Measles, Mumps, Newcastle disease, and Sendai viruses. All these viruses are well equipped for promoting highly specific and efficient malignant cell death, which can be direct and/or immuno-mediated. A number of proteins that serve as natural receptors for oncolytic paramyxoviruses are frequently overexpressed in malignant cells. Therefore, the preferential interaction of paramyxoviruses with malignant cells rather than with normal cells is promoted. Due to specific genetic defects of cancer cells in the interferon (IFN) and apoptotic pathways, viral replication has the potential to be promoted specifically in tumors. Viral mediation of syncytium formation (a polykaryonic structure) promotes intratumoral paramyxovirus replication and spreading, without exposure to host neutralizing antibodies. So, two related processes: efficient intratumoral infection spread as well as the consequent mass malignant cell death, both are enhanced. In general, the paramyxoviruses elicit strong anticancer innate and adaptive immune responses by triggering multiple danger signals. The paramyxoviruses are powerful inducers of IFN and other immuno-stimulating cytokines. These viruses efficiently promote anticancer activity of natural killer cells, dendritic cells, and cytotoxic T lymphocytes. Moreover, a neuraminidase (sialidase), a component of the viral envelope of Newcastle Disease, Mumps, and Sendai viruses, can cleave sialic acids on the surface of malignant cells thereby unmasking cancer antigens and exposing them to the immune system. These multiple mechanisms contribute to therapeutic efficacy of oncolytic paramyxoviruses and are responsible for encouraging results in preclinical and clinical studies.

## Introduction

Metastatic cancer remains largely an incurable disease which requires development of novel therapeutic strategies. While the accumulating knowledge on the molecular basis of cancer offers new potential targets for anticancer drugs, there is an alternative approach that relies on mechanisms developed through the millions of years of human coexistence with viruses. The viruses emerge as promising instruments against cancer. Substantial selectivity of infection and replication in cancer cells is characteristic of many viruses, and their therapeutic efficiency and safety can be dramatically improved by genetic manipulations.

Paramyxoviruses represent a well-studied model of oncolytic viruses with promising therapeutic potentials. The antitumor efficacy by oncolytic paramyxoviruses appears to be associated with three properties of the viruses: (i) selective replication in tumor cells, (ii) oncolytic properties by the viruses *per se*; and (iii) immunostimulatory capacity of the viruses. Studies on viral oncolysis mediated by paramyxoviruses have revealed some properties that collectively contribute to strong anticancer effects. Some of these properties are common to many oncolytic viruses and some are specific to oncolytic paramyxoviruses only. All these properties are described below.

## Biology of Paramyxoviruses

Paramyxoviruses (members of the *Paramyxoviridae* family) are associated with a number of diseases in animals and humans, such as measles virus (MV), mumps, and several respiratory infections. Sendai virus (SeV) affects mice and some other rodents. Newcastle disease virus (NDV) is associated with a contagious disease affecting many domestic and wild species of birds. SeV and NDV were not found to be associated with any serious human diseases, while MV and mumps are well-studied human pathogens. Few representatives of paramyxoviruses NDV, SeV as well as attenuated vaccine strains of MV and mumps viruses were tested as oncolytic agents in multiple model experiments and in a few clinical trials.

Paramyxoviruses enter the life cycle with binding of their attachment protein to an appropriate cell-surface receptor. There could be either direct fusion of the envelope with plasma membrane^1^ assisted by the F protein, which is activated by the interaction with sialic acid–containing surface glycoproteins,^2^ or the virus can enter the cell through the endocytic route with fusion occurring in acidic conditions inside endosomes.^3^ As a result, the nucleocapsid containing viral genome is released into the cytosol where viral replication takes place.

Viral RNA-dependent RNA polymerase transcribes the genes into mRNAs, which are then translated into structural and nonstructural proteins. The transcription starts from a single promoter located at the 3′ end of the genome and then may either terminate within specified regions between each viral gene or proceed further downstream. This mode of transcription is responsible for the observed product polarity in which the genes closest to the 3′ end of the genome are expressed more abundantly than their downstream counterparts.^1^

This mechanism is a simple and effective way for transcription level regulation to generate the necessary balance of viral products. A concentration of the most abundantly synthesized nucleoprotein determines the time at which RNA-dependent RNA polymerase switches from gene transcription to genome replication. Replication involves synthesis of full-length positive-strand RNAs, which are then transcribed into the progeny genomic minus-strand RNAs. The maturing virions finally gain their envelopes with the membrane-trapped viral glycoproteins by budding through the outer membrane. New virions can then infect other cells and enter new life cycles. An alternative pathway for spreading viral infection involves fusion of infected cells with their neighbors and formation of syncytia.^4^ Viral fusion proteins used by the virus to enter the cell are exposed to the cell surface of infected cells inducing fusion with plasma membranes of neighboring cells. Therefore, a single virion can potentially infect and kill dozens of cells.

## Three Levels of Cancer-Specific Infection and Spreading for Paramyxoviruses

### Receptors for paramyxoviruses are frequently overexpressed in malignant cells

**Figure 1:**
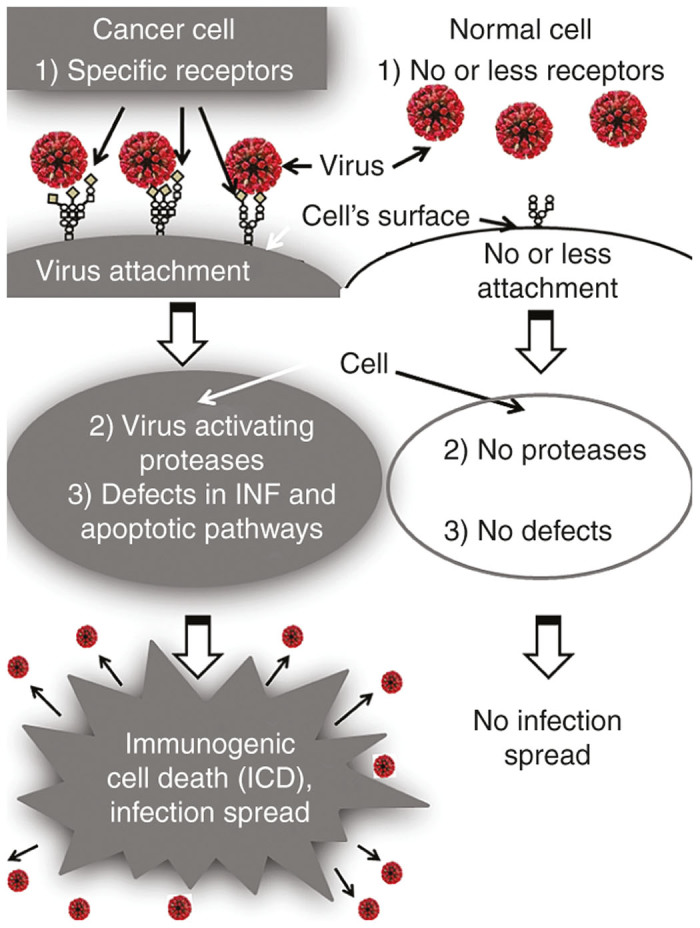
Three levels of cancer-specific infection spread for paramyxoviruses. (1) First level of virus specificity for cancer cells is related to overexpression of specific receptors for paramyxoviruses. Sialic acids residues in a form of sialoglycoproteins serve as receptors for NDV, mumps, and SeV. These sialoglycoproteins are frequently overexpressed in malignant cells. CD46 and nectin 4 serve as natural receptors for attenuated strain of MV, and they are also frequently overexpressed in malignant cells. (2) Second level of virus specificity is related to cancer cells’ specific proteases. The replication cycles of paramyxoviruses require protease cleavage of viral glycoproteins for productive cell enter. Desirable proteases for oncolytic viruses that can activate them are expressed preferentially by cancer cells. For example, matrix metalloproteinases are overexpressed in almost all human cancer cells. (3) Third level of virus specificity is related to cancer cells’ specific genetic defects that allow viral replication. During the process of malignization, a cancer cell accumulates many genetic changes. Along with mutations that promote accelerated proliferation and invasion, many cancerous cells are losing abilities to produce interferon (IFN) and to respond to IFN by apoptotic pathway induction. Such abnormalities make these cells highly susceptible to viral infection. So, oncolytic paramyxoviruses due to abundance of cancer-specific receptors and due to cancer-specific genetic defects are more likely spreading among malignant cells rather than among normal cells.

A first level of specificity for cancer cells is related to overexpression of specific receptors for oncolytic viruses (Figure 1). NDV, mumps, and SeV use sialic acid–containing sialoglycoproteins as the cell surface receptors.^5,6^ The abundant presence of sialoglycoproteins on the surface of cancer cells^7,8^ most likely promotes preferential association of the virus with malignant rather than with normal cells and contributes to their selective cytolytic effect in primary tumors and in metastases. Perhaps high sensitivity to SeV mediated cell death of sialic acid–rich prostate carcinoma cells, in comparison with normal prostate epithelium, is explained by SeV’s preferential association with malignant cells.^9^

The attenuated measles virus (Edmonston strain) (MVES) uses the CD46 receptor, which is a regulator of complement activation that is universally expressed in all nucleated human cells but is often overexpressed in tumor cells.^10^ MVES can kill cells that overexpress this receptor without significant cytopathic effect against nontransformed cells expressing low receptor levels.^11^ Nectin 4 has recently been identified as the additional receptor for MV.^12^ It is a member of adhesion receptors of the immunoglobulin superfamily localized to the adherents’ junctions of epithelial cells. Nectin 4 can be considered as a tumor cell marker for breast, lung, and ovarian cancers,^13–15^ suggesting that it can be partially responsible for the selectivity of measles virus toward cancer cells. Table 1 summarizes information related to viral receptors and their overexpression in malignant cells.

### Virus activation through cancer-specific proteases

A second level of cancer cells’ specificity is related to cancer cells’ specific proteases (Figure 1). The replication cycles of enveloped viruses, such as paramyxoviruses, require protease cleavage of viral glycoproteins for productive cell entry.^16^ Desirable proteases in cancer cells for oncolytic viruses should be expressed mainly by cancer cells. Such proteases exist; they are represented by matrix metalloproteinases.^17^ These molecules are endopeptidases that are overexpressed in almost all human cancer cells.^18–20^ It is still unknown whether these proteases can activate wild-type viruses. However, a recombinant Sendai virus,^21^ as well as MV constructs^22,23^ that are selectively activated in human tumor cells expressing matrix metalloproteinases, has been created.

### Genetic defects of cancer cells

A third level of cancer cells’ specificity is related to their specific genetic defects which allow viral replication (Figure 1). During the process of malignization, a cancer cell accumulates many genetic changes. Along with mutations that promote accelerated proliferation and invasion, many cancerous cells lose their ability to produce and respond to interferon (IFN) by apoptotic pathway induction.^24^ Such abnormalities make these cells highly susceptible to viral infection.^25^ So, due to the abundance of cancer-specific receptors, cancer-specific genetic defects, and proteases, oncolytic paramyxoviruses are more likely going to spread among malignant than among normal cells.

## Oncolysis Mediated by Direct Cancer Cell Killing through Formation of Syncytium

Paramyxoviruses have a mechanism of virus infection spread without the release of mature virus particles from the cells. The mechanism includes fusion of infected cells with their noninfected neighbors. The fusion results in the formation of a syncytium, a large polykaryonic conglomerate originating from many cells. A single infected cell could trigger up to 50–100 neighboring cells to fuse together and to form a syncytium.^26^ The process of cell fusion allows the virus to minimize exposure to host neutralizing antibodies. Usually, the syncytia survive no longer than 4–5 days.

Syncytium death is associated with nuclear fusion, premature chromosome condensation, and autophagic degeneration, accompanied by release of vesicles reminiscent of exosomes (syncytiosomes). “Dying syncytia produce significantly more syncytiosomes than normal cells or cells killed by irradiation, freeze thaw, or osmotic shock. These syncytiosomes also load dendritic cells (DCs) for antigen presentation more effectively than exosomes from cells dying by other mechanisms.”^27^

**Figure 2:**
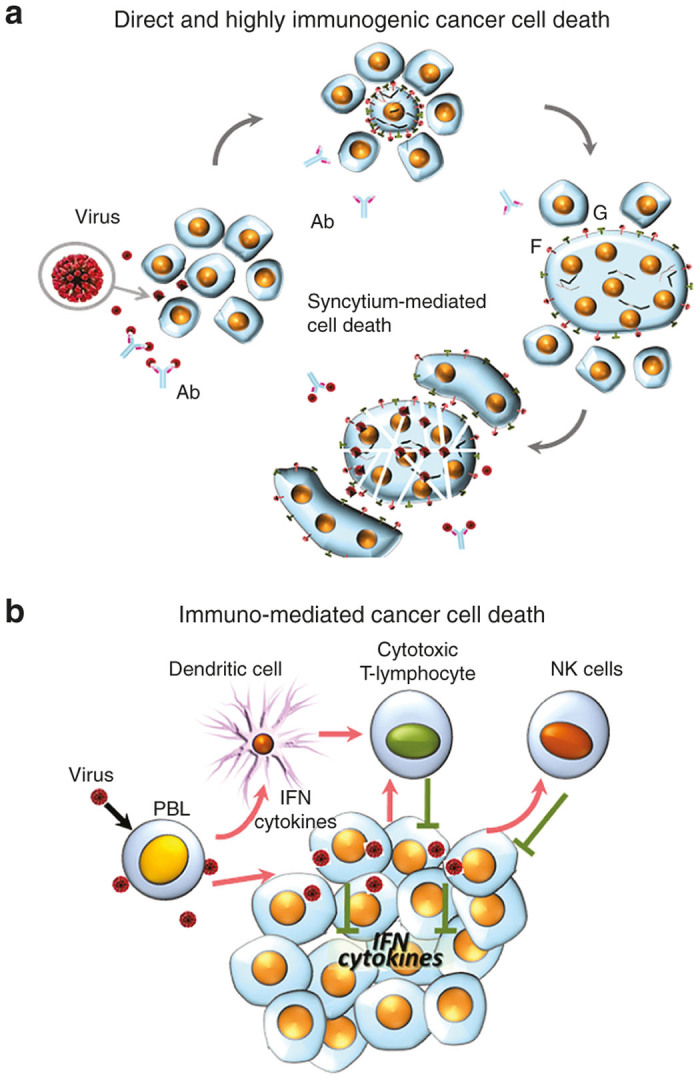
Direct or immuno-mediated cancer cells death. (**a**) Direct and highly immunogenic cancer cell death through formation of syncytia. Virus-infected cells start expressing viral fusion protein (F) at their cell surface that forces fusion of infected and surrounding noninfected cells and formation of large polykaryonic structures known as syncytia. The syncytia support continuous viral replication and further fusion with new uninfected cells. Eventually, the syncytia die. The syncytia formation allows viral infection to spread even in the presence of high titers of neutralizing antiviral antibodies. This type of cell death assists in viral oncolysis. Proteolytic cleavage of viral protein F0 is needed to activate it and transform it into F1. Only F1 can promote cell fusion into syncytia. For some representatives of paramyxoviruses, F0 that is expressed in infected human cells is fusion incompetent because of the absence of proteolytic cleavage to activate F-protein (F0) and transform it to F1. Therefore, syncytia formation could not expand in tumor tissues, unless they express a protease that could perform appropriate proteolytic cleavage. (**b**) Immuno-mediated cancer cell death. Paramyxoviruses stimulate innate and adaptive immunity that targets cancer cells. Malignant cells are frequently unable to react to virus infection by producing INF. They are also frequently unable to respond to INF by apoptotic pathway induction. In response to virus infection, normal cells are able to produce INF. Notably, virus induced INF is synthesized by macrophages, dendritic, endothelial and tumor stromal cells. Moreover, in response to virus infection, tumor stromal cells also secrete numerous cytokines that elicit a strong antitumor reaction by attracting macrophages, monocytes, natural killer (NK) cells and other components of the innate immune system. In addition, IFNs and cytokines stimulate dendritic cells (DCs) to educate cytotoxic T-lymphocytes to target tumor cells.

Viral fusogenic membrane glycoprotein expression causes syncytia development and consequent cellular immunogenic death,^4,28^ which can contribute to the efficiency of viral oncolysis. Consequently, the ability of viruses to induce the formation of syncytia might correlate with their oncolytic potential. This hypothesis is supported by some experimental evidence. The fusion protein of NDV was introduced into an oncolytic strain of vesicular stomatitis virus (VSV), with significant enhancement of oncolytic activity of the virus against multifocal hepatocellular carcinoma in the livers of immunocompetent rats.^29^ Similarly, the oncolytic activity of a strain of replication-competent herpes simplex virus (HSV) was significantly increased after the introduction of a hyperfusogenic glycoprotein from the gibbon ape leukemia virus.^30^ The fusogenic potential could be further increased by the introduction of amino acid substitutions. A novel NDV variant harboring an L289A substitution within the F gene possesses enhanced fusion and oncolytic activities against rat hepatocellular carcinoma cells, both *in vitro* and in immunocompetent rats.^31^ A mutant of SV5 paramyxovirus that harbors a glycine-to-alanine substitution in the fusion protein was hyperfusogenic and displayed an enhanced oncolytic activity.^32^ Remarkably, even plasmid vectors^25,33,34^ or replication-deficient viruses^35–37^ expressing the fusogenic membrane glycoproteins are capable of promoting significant tumor regression, suggesting that cell fusion and syncytia formation substantially contribute to the oncolytic activity of paramyxoviruses. So, ability to kill cancer cells directly through formation of syncytium is an important property of oncolytic paramyxoviruses (Figure 2a).

## Summary of Factors that Affect Virus Production Level in Malignant Cell

It is noticeable from the sections presented above that the factors potentially influencing paramyxovirus production levels in cancer cells are virus and cancer cell specific. So, a paramyxovirus representative and a cancer cell have to complement each other to achieve effective virus-mediated tumor destruction. A particular virus receptor should be overexpressed in a particular malignant cell for efficient virus attachment. A malignant cell should have genetic defects in IFN and apoptotic pathways that allow viral replication in this cell. A malignant cell should overexpress a specific protease for efficient viral particle maturation. The F protein of the paramyxovirus utilized should have a suitable proteolytic cleavage site for the malignant cell-specific protease to enable virus processing and maturation. The viral F protein should be able to induce malignant cell fusion (syncytium formation) that allows paramyxoviruses to minimize exposure to host neutralizing antibodies and maximize yield of infectious particles. Through genetic engineering, it is possible to create viral constructs with higher affinity to malignant cells receptor (described below in the section Improving tumor-specific targeting), constructs with better proteolytic processing abilities by cancer cells specific matrix metalloproteinases and constructs with better cancer cell fusing activities (described in the section *Optimization of syncytia formation and F protein cleavage site*). However, it is very difficult to optimize all the factors in one genetic construct for most efficient malignant cells attachment, replication, and particles maturation but perhaps it will be accomplished in future.

## Oncolysis Mediated by Paramyxovirus-Induced Stimulation of the Antitumor Immune Response

In addition to the direct killing of infected cancer cells, viral infection elicits systemic responses that strongly contribute to viral oncolysis. These responses include stimulation of antiviral innate immunity, such as the production of IFNs, other cytokines, and the activation of natural killer (NK) cells. Also, viral infection elicits adaptive immune responses that not only act to contain the infection by targeting the released viral particles and the infected cells, but also assist in the exposure of cancer cells to the antigen-presenting cells leading to activation of the anticancer immune response (Figure 2b).

### Enhancement of cancer cell immunogenicity

Paramyxoviruses, including oncolytic paramyxoviruses, often modulate genes related to antigen presentation and enhance the immunogenicity of cancer cells. For example, it has been known that NDV upregulates human lymphocyte antigens (HLA) class I and II as well as costimulatory molecule intercellular adhesion molecule 1 in human cancer cells.^38^

### Contribution of danger signals

Oncolytic viruses could play a crucial role in tumor rejection by helping to overcome immune suppression mediated by the tumor microenvironment. Perhaps, to some degree, anticancer immune activation can occur even without replication of a virus in a tumor mass. It is likely the antitumor immune response can be triggered even if the virus is injected into skin or intratumoral.^39^ By its nature, the virus acts as a danger signal, which is able to recruit and activate professional antigen-presenting cells such as DCs. DCs act as part of both innate and adaptive immunity and can prime adaptive T cell immune responses against specific, tumor-associated antigens (TAAs).^40^ Paramyxoviral fusogenic membrane glycoproteins promote syncytia formation that triggers particular efficient TAA presentation by DC.^27^

In general, DC activation can be induced by endogenous and exogenous danger signals.^41^ In current terms, the endogenous signals are damage-associated molecular patterns (DAMPs) and exogenous danger signals are pathogen-associated molecular patterns (PAMPs) molecules. DAMPs and PAMPs act to initiate immune responses to infection, pathological processes, and therapeutic processes such as oncolytic virotherapy.^42–44^ Cancer cells can evade the immune system by conditioning their microenvironment until viruses overcome the immunosuppressive environment. They produce immunosuppressive cytokines and recruit immunosuppressive cells, such as T regulatory cells (T-regs), myeloid derived suppressor cells, and M2 macrophages to maintain immune tolerance within the tumor.^45^ Acting on both cancer and immune cells, oncolytic paramyxoviruses elicit potent adaptive immunity against specific tumor antigens and thus against cancer cells. These viruses operate as immuno-adjuvants to overcome the immunosuppressive tumor microenvironment, as cell-surface exposed viral antigens and products of dying cells provide potent danger signals. As a result, immunogenic cell death (ICD) of cancer cells gets promoted. It may resemble apoptosis, necrosis, pyroptosis, or autophagic cell death.^46,47^ DAMPs include the calreticulin (ecto-CRT) and heat-shock proteins (HSP70, HSP90) exposed on the cell surface, high-mobility group box 1 (HMGB1) protein, ATP, and uric acid released from dying cells. This release happens during mid to late phases of apoptosis, necrosis, or autophagy.

Introduction of PAMPs to cancer cells through virus infection is related to the previously described processes known as artificial heterogenization^48^ or xenogenization^49^ meaning that pathogens increase cancer cells visibility for immune system. The danger signals activate DCs, which cross-present TAAs to cluster of differentiation (CD) 8^+^ T cells for potent antitumor immunity. By this mechanism, MV induces ICD in human melanoma.^50^ Following ICD of cancer cells infected with MV vaccine, the apoptotic bodies are ingested by DCs, which mature and then produce high levels of IFN-α, and cross-present TAAs, leading to a generation of tumor-specific CD8^+^ cytotoxic T cells.^51,52^

### Role of autophagy

Autophagy is a well-regulated process that mediates degradation and recycling of cellular proteins, organelles, as well as pathogens.^53^ Autophagy plays important roles in both innate and adaptive immunity.^54,55^ A number of paramyxoviruses, including MV,^56^ NDV,^57^ and SeV,^53^ have been shown to induce autophagy in infected cancer or normal cells. As mentioned earlier, death of syncytia is associated with autophagy.^58^ Interestingly, the engagement of CD46, a ubiquitous human surface receptor, which is able to bind several different pathogens including MV, is sufficient to induce autophagy.^59^ Autophagy enhances tumor immunogenicity not only by releasing DAMPs but also by promoting an antigen cross-presentation from cancer cells to DCs and then to T cells. Cross-presentation of TAAs by DCs is promoted by the nonapoptotic (most likely autophagic) cell death induced by paramyxoviral fusogenic membrane glycoproteins.^27^

### Inhibition of the IFN response in target cells

One of the most important defense mechanism a cell has against viral infection is induction of IFNs and IFN-inducible proteins, resulting in suppression of protein synthesis and establishment of an antiviral state. However, viruses have evolved diverse strategies to evade or antagonize the IFN antiviral response proteins. Paramyxoviruses encode and use a number of proteins, which facilitate escape from cellular defense by blocking IFN production or/and signaling. NDV and mumps viruses use V- protein,^60,61^ SeV uses a family of C-protein,^61^ V-protein,^62^ and MV uses V-protein,^63^ P-protein,^64^ and C-protein^65^ to block the IFN pathway.

During a process of malignization, a tumor cell genetically changes and gets rid of functions that serve the needs of the whole organism. Along with mutations that promote accelerated proliferation and invasion, the IFNs signaling is often compromised through a homozygous loss of the chromosome 9p21 region, which encodes type I IFN genes.^24^ In malignant cells, virus may not affect IFN signaling as this pathway is already broken. However, sometimes during the genetic engineering process, it is necessary to minimize the potential impact of viral construct on healthy cells; therefore, viral genes, which block the IFN pathway, are deleted or attenuated.^66^

### Stimulation of the IFN response in peripheral blood leukocytes

Paramyxoviruses are known to be strong inducers of IFNs in human peripheral blood leukocytes (HPBLs). This property that has been used in biotechnology: the Sendai virus was chosen from among many other viruses for industrial-scale production of IFN from human leukocytes.^67^ In HPBLs, SeV behaves as a potent inducer of IFN-α^68^; it induces at least nine different IFN-α species.^69^ In HPBLs, SeV also stimulates the production of IFN-γ.^70^ Similarly, the NDV stimulates the production of IFN-α from several gene isoforms.^71^ The IFN secretion can be induced by the viral Hemagglutinin-Neuraminidase (HN-protein or HN), a process independent of the double-stranded RNA (dsRNA) response, and by dsRNA replicative intermediates.^71,72^ Perhaps, the existence of two alternative parallel mechanisms of IFN induction explains the high IFN stimulating capacity of NDV^72,73^ and other paramyxoviruses.

IFNs exert complex systemic effects that make them a useful means for adjuvant therapy of cancer.^74,75^ IFNs have been used in therapeutic schemes for the treatment of metastatic melanoma, renal carcinoma, Kaposi sarcoma, bladder carcinoma, hairy-cell leukemia, etc. The significant prolongation of a disease-free survival has now been largely validated by the combined analyses of multi-institutional trials and by the subsequent studies that have included meta-analyses.^76^ IFNs can act systemically by regulating the immune response through the activation of DCs, cytotoxic T cells, and NK cells. IFNs markedly increase the major histocompatibility complex (MHC) class I and class II-dependent antigen presentation that causes increased presentation of tumor-specific antigens by malignant and professional antigen-presenting cells. This presentation stimulates antitumor activity and proliferation of cytotoxic T lymphocytes.^77–79^

The responses to IFNs are dysfunctional in many malignancies making the direct effects of IFNs less efficient. However, IFNs can affect tumor growth indirectly, through suppression of angiogenesis by altering the stimuli from tumor cells and by inhibiting endothelial cells. The degree of inhibition correlates with the reduced tumor vascularization and the consequent retardation of tumor growth.^76,80,81^ The treatment with IFNs may also affect the viability of cancer stem cells that are highly resistant to chemo- and radiotherapy and are responsible for the disease relapses. Most likely, IFNs affect tumor vasculature and disrupt the vascular niche of stem cells, as it was found in murine xenografts of human glioma.^75^ It is suggested that IFN-γ triggered by ultraviolet (UV) inactivated SeV is responsible for lung metastasis suppression in melanoma murine model.^82^

### Stimulation of cytokines

In HPBLs, SeV was shown to stimulate the synthesis of interleukins (IL) 2, 6, 8, macrophage inflammatory protein-1 (MIP-1) α, MIP-1-β, regulated on activation normal T cell expressed and secreted (RANTES) proteins, and monocyte chemotactic protein -1 (MCP-1).^68,70^ Live or UV-irradiated SeV can stimulate the IL-6 release in treated animals.^83^ In DCs, incubation with SeV results in the IL-6 secretion; the fusion protein (F) was found to be responsible for the effect.^84^ An injection of an UV-inactivated SeV into established mouse renal cell carcinoma tumors resulted in the secretion of chemokine C-X-C motif chemokine 10 (CXCL10) by infiltrating DCs.^85^ NDV was also shown to induce secretion of chemokine (C-C motif) ligand 5 (CCL5) (also known as RANTES) and CXCL10 from infected human cancer cells.^38^ The CXCL10 was found to have an antitumor activity through the attraction of monocytes, macrophages, NK cells, T cells, and DCs. The CXCL10 also promotes T cells adhesion to endothelial cells and the inhibition of angiogenesis.^86^ CCL5 and CXCL10 are T helper type 1 (Th1) chemokines and can attract activated Th1 and TCL cells into tumor nodules to exert their cytotoxicity on cancer cells.^87^ In HPBLs, incubation with NDV also stimulates the secretion of cytokines such as tumor necrosis factor α (TNF-α)^88,89^ and TNF-related apoptosis-inducing ligand (TRAIL).^71,89^ It appears that the induction of TRAIL is due to a single viral HN protein.^71,90^ NDV induces the secretion of TRAIL in monocytes and NDV kills various human tumor cell lines following the stimulation of TRAIL receptors 2 (TRAIL-R2).^90^ Macrophages can also be stimulated by incubation with NDV leading to upregulation of a set of macrophage-specific genes and secretion of TNF-α.^91^

### Stimulation of NK cells

NK cells constitute a type of cytotoxic lymphocytes that bridge the innate and adaptive branches of the immune system. These cells widely interact with other components of the immune system affecting the antigen-specific T and B cell responses.^92^ The NK cells participate in the early control against virus infection and in tumor immune surveillance. These cells are responsible for a fast protective effect due to their ability to destroy abnormal or stressed cells that lack antigen-specific cell surface receptors and MHC. The NK cells do not require activation in order to kill cells, which are not expressing “self” markers of the MHC class I.

The NK cells can be activated for most efficient NK-mediated cell lysis. NK cells display natural cytotoxicity receptors that are responsible for their activation. Among these receptors are NK protein 46 (NKp46) and NK protein 44 (NKp44). Hemagglutinin neuraminidase (HN) protein of paramyxoviruses directly interacts with two receptors types: NKp44 and NKp46, and this interaction triggers the NK activation and NK-mediated cell lysis.^93–96^ In particular mumps viral HN protein mediates NKp46 activation of NK cells, and this activation results in tumor rejection via NK-DC crosstalk.^97^ Studies with the UV-inactivated SeV virus also highlight the important role of NK cells in virus-mediated oncolysis. In a mouse renal cancer model, UV-inactivated SeV produced strong anticancer effect, which was compromised by coinjection of an NK-depleting anti- mono-sialo-tetra-hexosyl-ganglioside 1 (GM1) antibody.^85^

### Stimulation of DCs

DCs are professional antigen-presenting cells that can efficiently stimulate innate as well as adaptive immune responses against various pathogens and cancer cells. After sensing a virus or other pathogens, the DCs enter a maturation program and became competent for education of naive T cells.

The *ex vivo* infection of DCs by recombinant SeV can induce DCs maturation and activation in only 1 hour.^98^ Treatment with activated DCs harboring different variants of recombinant SeV can dramatically improve the survival of animals inoculated with cells of malignant melanoma,^82,99^ colon carcinoma,^100^ squamous cell carcinoma,^101^ hepatocellular carcinoma, neuroblastoma, and prostatic cancer.^102^ The administration of such DCs before tumor inoculation has demonstrated a preventive effect against lung metastasis of neuroblastoma^103^ and prostate adenocarcinoma.^104^ Another response of DC cells to virus infection is inhibition of immunosuppression mediated by the regulatory T-cells. In experiments with the UV-inactivated SeV, carbohydrates of the viral fusion (F) protein are recognized by an unknown receptor(s) on DC cells leading to the nuclear factor kappa-light-chain-enhancer (NF-kB) activation of B cells and secretion of IL-6.^84^

### Stimulation of tumor-specific cytotoxic T-cell mediated activity

Cell surface binding of NDV can stimulate the tumor-specific cytotoxic CD8+ T-cell (CTL) response and increase CD4+ T-helper cells’ activity even in the absence of an antiviral T-cell response and cell infection.^105^ The activation and infiltration of tumor sites with CTLs is the result of complex effects of virus triggered immune system, including local and systemic release of IFNs and other cytokines. Interestingly, UV-inactivated NDV was as active in promoting the tumor-specific CTL response as the replication competent NDV. It was found that this effect is mediated by the presence on the tumor cell surface of functional viral HN molecules that have affinity to plasma membranes and therefore can mediate a strong adhesion of the infected cells to CTLs.^105^ Since the HN proteins of many paramyxoviruses are homologous, it is likely that the HN protein, regardless of whether it derives from NDV or other related paramyxoviruses, can activate both NK and CTL responses.

### Viral sialidase activity of the HN protein

Metastatic cancer cells often express a high density of sialic acid-rich glycoproteins that increase the invasive potential.^106^ The overexpression of sialic acid on the surfaces creates a negative charge on cell membranes, leading to repulsion between cells that promote metastases by helping cancer cells entry into the blood stream. Indeed, the ability of tumor cells to metastasize correlates with the abundance of sialic acids on the surface of many types of malignant cells.^106–108^ The extent of cell surface poly-sialylation was suggested as a marker characterizing the differentiation status of thyroid and small-cell lung carcinoma cells.^109^

A more detailed analysis reveals qualitative differences in sialic acids on the surface of cells displaying different degrees of invasiveness. While in normal human colon cells, as well as in adenomas and several carcinomas of different grades, an alpha 2, 3-linked sialic acid is detected, in the highly malignant variants, alpha 2, 6-linked sialic acids are also present. It was found that the malignant progression was associated with the *de novo* expression of an alpha 2,6 sialyl-transferase, which transforms the alpha 2,3-linked sialic acid into the 2,6-linked sialic acid.^110^ The increase in alpha 2,6 sialylation coincident with tumor progression is detected for hepatocellular^111^ and colon carcinomas.^112^

**Figure 3:**
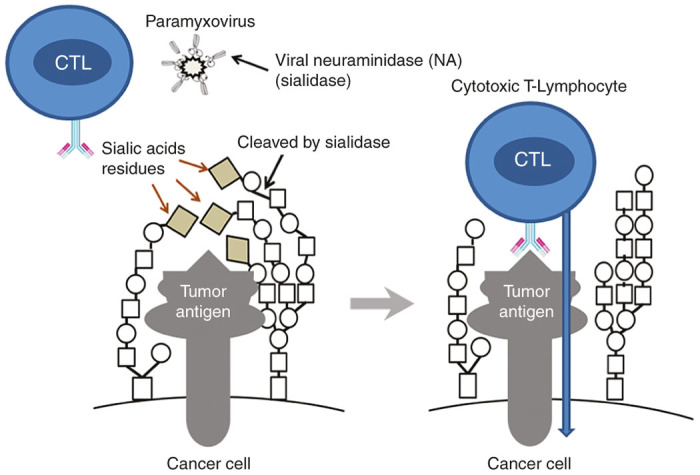
A hypothetical role of viral sialidase in activation of cytotoxic T-lymphocyte response against cancer cells. The removal of sialic acids may unmask hidden tumor antigens and make them more visible by the immune system.

Inhibitors of the sialylation process decrease the malignancy of cancer cells and now are considered as candidate drugs against metastatic cancer.^113^ One of the possible mechanisms linking the increased sialylation with malignant phenotype is the creation of a thick “coat” on the cell surface that hides cancer antigens and provides an escape of malignant cells from the immunosurveillance. For example, it is found that the tumor specific Lewis antigen of malignant medullary thyroid cancer can be masked from the immune system by both alpha 2,3- and alpha 2,6-linked sialic acid residues.^114^ Removing some sialic acid residues from the surface of malignant cells by sialidase can unmask cancer-specific antigens and make cells visible to the immune system (Figure 3). The removal of sialic acids from tumor cells is associated with a reduced growth potential, an activation of NK cells, and a secretion of IFN-γ.^115^

The HN proteins present in SeV, NDV, and some other paramyxoviruses possess both an erythrocyte agglutination and a neuraminidase (sialidase) activities.^116,117^ Neuraminidase is capable of cleaving and removing sialic acid residues from the surface of malignant cells leading to a dramatic increase in their ability to induce the T-cell response.^118^

## Summary of Anticancer Immuno-Triggering Abilities of Oncolytic Paramyxoviruses

A number of molecular mechanisms are responsible for the anticancer immuno-triggering abilities of oncolytic paramyxoviruses. One of them is related to viral modulation of gene expression related to antigen presentation that results in enhancement of the immunogenicity of cancer cells. Another is related to induction of autophagy in infected cancer cell that also enhances cancer cells immunogenicity. Viral sialidase activity of paramyxovirus HN protein might also contribute to improvement of cancer cell visibility for immune system by unmasking cancer antigens from a “thick coat” of sialic acid polymers. In addition, oncolytic paramyxoviruses stimulate anticancer NK cells and tumor-specific cytotoxic T cell–mediated activity. They also trigger activation of DCs, which are responsible for better cancer antigen presentation.

Strong immune activation by paramyxoviruses is a double edged sword. On one hand, it triggers strong anticancer immunity. On the other hand, it can inhibit replication of paramyxoviruses and trigger their fast clearance from a patient’s body. So, perhaps a panel of different oncolytic viruses that represents different families instead of one particular virus should be used in a sequential way for maximizing a chance of complete remission during a course of oncolytic virotherapy.

## Comparison of Oncolytic Paramyxoviruses with Other Oncolytic Viruses

Many oncolytic viruses that belong to various viral families are currently being tested in clinical trials.^17,119^ It is becoming clear that oncolytic effects can be produced by most animal viruses having quite different genome organizations: from small positive-strand RNA-containing picornaviruses,^120–123^ small double-stranded (ds) RNA reoviruses,^124^ small ds-DNA parvoviruses,^125^ to medium-size ds-DNA adeno-^126,127^ and herpes viruses,^128^ negative-strand RNA rhabdo-^129^ and paramyxoviruses^73,130^ as well as very large poxviruses.^131,132^ The high degree of diversity among these agents suggests that while there could be common mechanisms of oncolysis characteristic to all viruses, certain specific mechanisms could be associated with particular groups of viruses.

Are there any advantages in using paramyxoviruses versus representatives of other viral families as oncolytic agents? We believe that the ability to trigger syncytium formation is a great advantage of oncolytic paramyxoviruses. As mentioned earlier (see section *Oncolysis mediated by direct cancer cell killing through formation of syncytium*), paramyxoviruses have a mechanism of spreading infection without the release of mature virus particles from cells. This includes fusion of infected cells with their noninfected neighbors. The fusion results in the formation of a syncytium from 50–100 neighboring cells (Figure 2).^26^ The ability of the viruses to induce the formation of syncytia correlates with their oncolytic potential. Most likely, syncytia formation contributes to the efficiency of viral oncolysis because it allows extra rounds of viral replication without any exposure to host neutralizing antibodies. Moreover, malignant cells death through syncytia fusion is highly immunogenic and resembles autophagy (see section **“**role of autophagy”). Fused tumor cells secrete an abundance of “syncytiosomes,” which are exosome-like vesicles that highly efficiently present TAA via MHC molecules.^27,33^ In addition, cross-presentation of TAAs by DCs is strongly promoted by paramyxoviral fusogenic membrane glycoproteins.^27^

Another potential unique advantage of some paramyxoviruses (Mumps, NDV, and SeV) is the sialidase (neuraminidase) activity of their HN protein which has the potential to remove sialic residues from the surface of tumor cells (see section *Viral sialidase activity of the HN protein*). Such removal is associated with reduced growth potential of malignant cells, activation of NK cells, and secretion of IFN-γ.^115^

## Future Perspectives

### Improving tumor-specific targeting

NDV, SeV, and Mumps viruses can target a variety of cancers because they use sialic acids residues as their cell receptors. However, these viruses are also able to bind sialic acid residues on normal cells. So, antibodies that diminish viral binding to normal cells and increase viral binding to malignant cells could be very useful for improving target specificity. Such bi-specific antibodies have been developed for NDV. These antibodies indeed increase NDV target tumor cells specificity in animal model experiments.^133^

A number of studies for increasing target specificity were also performed with MVES. Even though CD46 and nectin 4 receptor binding already provides some malignant cell target specificity for MVES, it could be further increased through variable routes described below.

A series of studies on retargeting oncolytic strains of MVES employed viral attachment hemagglutinin (H) protein,^134^ which is responsible for the receptor recognition and the binding specificity.^135^ To kill interaction of the H-protein with CD46, the region responsible for the binding is mutagenized, and a variety of ligands that bind to selected targets of potential host cells are introduced. The ligands successfully tested in this system include the antigen-binding peptides (single-chain antibodies) against epidermal growth factor receptor (EGFR; synonyms ErbB-1; HER1 in humans),^136–138^ HER-2/neu,^139^ folate receptor α,^140^ CD20,^141^ CD38,^137^ a peptide echistatin that binds to integrins,^142^ and cytokine IL-13.^143^

### Optimization of syncytia formation and F protein cleavage site

Ability of paramyxoviruses to form syncytia increases viral oncolytic potency. The F protein of paramyxoviruses is responsible for viral fusion with the cell membrane. It is also responsible for viral spread from cell to cell via formation of syncytia. The F protein cleavage site is a determinant of virulence for different NDV viral strains.^144,145^ The introduction of the multibasic cleavage site into the F protein allows this protein to be cleaved and to be activated by a broad range of proteases. To increase oncolytic potency of one NDV strain, a polybasic cleavage site was introduced into the F protein to generate a new site. While the resultant virus exhibited only an intermediate virulence phenotype based on a mean death time in embryonated eggs, the virus formed large syncytia and was enhanced in its replication in cancer cells, leading to enhanced oncolytic effects in various animal tumor models.^146–152^

Similar findings were shown when the F protein of the NDV La Sota strain was modified in an analogous fashion.^144,153^ “The fusogenic and oncolytic activity of the rNDV/F3aa strain was further enhanced by a point mutation in the F protein at residue 289 from leucine to alanine, generating rNDV/F3aa (L289A). In an immunocompetent liver tumor rat model, administration of the mutant virus via hepatic arterial infusion resulted in improved syncytia formation and necrosis. This improvement in syncytia formation caused a significant 20% prolongation of survival.”^31,146^

Introduction of specific cleavage sites into the F protein allows utilization of tumor tissue-specific proteases, as demonstrated with SeV.^21,154,155^ Introduction of a ubiquitously recognized cleavage-motive into the F protein of SeV enables its continuous spread in malignant human tissues. A tumor-specific spreading of such recombinant SeV without evidence of bio-distribution into nonmalignant tissues^66^ was found in a hepatoma xenograft animal model. Such viral constructs represent candidates for clinical trials.

### Combination with autologous cell vaccines: a personalized oncolytic viral immunotherapy

“A therapeutic, autologous, antigen-presenting cell vaccine (Sipuleucel-T) was recently approved by the US Food and Drug Administration for metastatic prostate cancer, after demonstration of prolongation of overall survival in a randomized, double-blind, placebo-controlled study”.^156^ Combination of autologous vaccine with an oncolytic paramyxovirus could provide extra benefit for specific antitumor immune stimulation. The use of autologous tumor tissue for development of a personalized oncolytic viral immunotherapy should be further exploited with oncolytic paramyxoviruses.

### Individual viral sensitivity test

There is a growing interest in personalized cancer therapy, which involves identifying those treatments which may work best for an individual’s cancer. Individual viral sensitivity testing should involve testing an individual’s cancer cells in the laboratory to see which virus demonstrate the best response. It therefore could provide guidance about which oncolytic virus representative may be best for the individual in clinical practice.

## Conclusions

The described mechanisms of oncolysis mediated by paramyxoviruses indicate that these viruses represent exceptionally attractive instruments for cancer therapy. The genomes of paramyxoviruses are comparatively stable. The viruses induce efficient syncytium-mediated lysis of cancer cells and they elicit strong immunomodulatory effects, which dramatically enforce immune surveillance and anticancer resistance. The use of nonhuman paramyxoviruses, such as NDV, or SeV, or attenuated vaccine strains of MV, or Mumps viruses hold great advantage, as these virus strains are not associated with any serious pathology in humans.

Fast screening tests of biopsy materials are needed because variable malignancies might support a particular virus representative reproduction to a different level. Such tests should reveal which virus kills a particular malignancy in the most efficient way. In other words, an experimentally verified matching of a particular malignancy with a particular virus is highly desirable. In case several different virus representatives (perhaps even from a few different viral families) would be found to be efficient for one type of malignancy, they all might be used for virotherapy in a sequential or parallel way. Because of the potential for fast immune-mediated clearance of a particular virus from a patient, the sequential therapy may ameliorate treatment efficiency decline due to such clearance.

## Figures and Tables

**Table 1:** Oncolytic paramyxoviruses and their receptors overexpression in cancer cells

*Virus*	*Receptor*	*Receptor overexpression*
Newcastle disease virus (NDV)	Syalic acid containing molecules^2^	Invasive and metastatic cancers^8^
Sendai virus (SeV)		
Mumps		
Measles virus Edmonston strain (MVES)	CD46 (ref. 11)	Breast and ovarian cancers and other malignancies^157,158^
	Nectin 4 (refs. 12,159)	Breast, lung, and ovarian cancers^13,14,160^

## References

[bib1] Lamb, RA and Parks, GD (2007). Paramyxoviridae: the viruses and their replication. In: Knipe, DM and Howley PM (eds). Fields Virology, 5th edn. Lippincott Williams & Wilkins: Philadelphia. pp. 1449–1496.

[bib2] Villar, E and Barroso, IM (2006). Role of sialic acid-containing molecules in paramyxovirus entry into the host cell: a minireview. Glycoconj J 23: 5–17. 16575518 10.1007/s10719-006-5433-0

[bib3] Cantín, C, Holguera, J, Ferreira, L, Villar, E and Muñoz-Barroso, I (2007). Newcastle disease virus may enter cells by caveolae-mediated endocytosis. J Gen Virol 88(Pt 2): 559–569. 17251575 10.1099/vir.0.82150-0

[bib4] Higuchi, H, Bronk, SF, Bateman, A, Harrington, K, Vile, RG and Gores, GJ (2000). Viral fusogenic membrane glycoprotein expression causes syncytia formation with bioenergetic cell death: implications for gene therapy. Cancer Res 60: 6396–6402. 11103804

[bib5] Bossart, KN, Fusco, DL and Broder, CC (2013). Paramyxovirus entry. Adv Exp Med Biol 790: 95–127. 23884588 10.1007/978-1-4614-7651-1_6 PMC8782154

[bib6] Matrosovich, M, Herrler, G and Klenk, HD (2013). Sialic acid receptors of viruses. Top Curr Chem (epub ahead of print). 10.1007/128_2013_466 PMC7120183 23873408

[bib7] Büll, C, Stoel, MA, den Brok, MH and Adema, GJ (2014). Sialic acids sweeten a tumor’s life. Cancer Res 74: 3199–3204. 24830719 10.1158/0008-5472.CAN-14-0728

[bib8] Büll, C, den Brok, MH and Adema, GJ (2014). Sweet escape: sialic acids in tumor immune evasion. Biochim Biophys Acta 1846: 238–246. 25026312 10.1016/j.bbcan.2014.07.005

[bib9] Kawaguchi, Y, Miyamoto, Y, Inoue, T and Kaneda, Y (2009). Efficient eradication of hormone-resistant human prostate cancers by inactivated Sendai virus particle. Int J Cancer 124: 2478–2487. 19173282 10.1002/ijc.24234

[bib10] Myers, R, Greiner, S, Harvey, M, Soeffker, D, Frenzke, M, Abraham, K et al. (2005). Oncolytic activities of approved mumps and measles vaccines for therapy of ovarian cancer. Cancer Gene Ther 12: 593–599. 15746945 10.1038/sj.cgt.7700823

[bib11] Anderson, BD, Nakamura, T, Russell, SJ and Peng, KW (2004). High CD46 receptor density determines preferential killing of tumor cells by oncolytic measles virus. Cancer Res 64: 4919–4926. 15256464 10.1158/0008-5472.CAN-04-0884

[bib12] Noyce, RS, Bondre, DG, Ha, MN, Lin, LT, Sisson, G, Tsao, MS et al. (2011). Tumor cell marker PVRL4 (nectin 4) is an epithelial cell receptor for measles virus. PLoS Pathog 7: e1002240. 21901103 10.1371/journal.ppat.1002240 PMC3161989

[bib13] Takano, A, Ishikawa, N, Nishino, R, Masuda, K, Yasui, W, Inai, K et al. (2009). Identification of nectin-4 oncoprotein as a diagnostic and therapeutic target for lung cancer. Cancer Res 69: 6694–6703. 19679554 10.1158/0008-5472.CAN-09-0016

[bib14] Derycke, MS, Pambuccian, SE, Gilks, CB, Kalloger, SE, Ghidouche, A, Lopez, M et al. (2010). Nectin 4 overexpression in ovarian cancer tissues and serum: potential role as a serum biomarker. Am J Clin Pathol 134: 835–845. 20959669 10.1309/AJCPGXK0FR4MHIHB PMC3042138

[bib15] Fabre-Lafay, S, Monville, F, Garrido-Urbani, S, Berruyer-Pouyet, C, Ginestier, C, Reymond, N et al. (2007). Nectin-4 is a new histological and serological tumor associated marker for breast cancer. BMC Cancer 7: 73. 17474988 10.1186/1471-2407-7-73 PMC1868744

[bib16] Cattaneo, R (2010). Paramyxovirus entry and targeted vectors for cancer therapy. PLoS Pathog 6: e1000973. 20585633 10.1371/journal.ppat.1000973 PMC2891830

[bib17] Cattaneo, R, Miest, T, Shashkova, EV and Barry, MA (2008). Reprogrammed viruses as cancer therapeutics: targeted, armed and shielded. Nat Rev Microbiol 6: 529–540. 18552863 10.1038/nrmicro1927 PMC3947522

[bib18] Egeblad, M and Werb, Z (2002). New functions for the matrix metalloproteinases in cancer progression. Nat Rev Cancer 2: 161–174. 11990853 10.1038/nrc745

[bib19] Nagini, S (2012). RECKing MMP: relevance of reversion-inducing cysteine-rich protein with kazal motifs as a prognostic marker and therapeutic target for cancer (a review). Anticancer Agents Med Chem 12: 718–725. 22292753 10.2174/187152012802650237

[bib20] Hadler-Olsen, E, Winberg, JO and Uhlin-Hansen, L (2013). Matrix metalloproteinases in cancer: their value as diagnostic and prognostic markers and therapeutic targets. Tumour Biol 34: 2041–2051. 23681802 10.1007/s13277-013-0842-8

[bib21] Kinoh, H, Inoue, M, Washizawa, K, Yamamoto, T, Fujikawa, S, Tokusumi, Y et al. (2004). Generation of a recombinant Sendai virus that is selectively activated and lyses human tumor cells expressing matrix metalloproteinases. Gene Ther 11: 1137–1145. 15085175 10.1038/sj.gt.3302272

[bib22] Springfeld, C, von Messling, V, Frenzke, M, Ungerechts, G, Buchholz, CJ and Cattaneo, R (2006). Oncolytic efficacy and enhanced safety of measles virus activated by tumor-secreted matrix metalloproteinases. Cancer Res 66: 7694–7700. 16885371 10.1158/0008-5472.CAN-06-0538

[bib23] Mühlebach, MD, Schaser, T, Zimmermann, M, Armeanu, S, Hanschmann, KM, Cattaneo, R et al. (2010). Liver cancer protease activity profiles support therapeutic options with matrix metalloproteinase-activatable oncolytic measles virus. Cancer Res 70: 7620–7629. 20858718 10.1158/0008-5472.CAN-09-4650

[bib24] Haus, O (2000). The genes of interferons and interferon-related factors: localization and relationships with chromosome aberrations in cancer. Arch Immunol Ther Exp (Warsz) 48: 95–100. 10807049

[bib25] Mansour, M, Palese, P and Zamarin, D (2011). Oncolytic specificity of Newcastle disease virus is mediated by selectivity for apoptosis-resistant cells. J Virol 85: 6015–6023. 21471241 10.1128/JVI.01537-10 PMC3126310

[bib26] Galanis, E (2010). Therapeutic potential of oncolytic measles virus: promises and challenges. Clin Pharmacol Ther 88: 620–625. 20881957 10.1038/clpt.2010.211

[bib27] Bateman, AR, Harrington, KJ, Kottke, T, Ahmed, A, Melcher, AA, Gough, MJ et al. (2002). Viral fusogenic membrane glycoproteins kill solid tumor cells by nonapoptotic mechanisms that promote cross presentation of tumor antigens by dendritic cells. Cancer Res 62: 6566–6578. 12438252

[bib28] Lin, EH, Salon, C, Brambilla, E, Lavillette, D, Szecsi, J, Cosset, FL et al. (2010). Fusogenic membrane glycoproteins induce syncytia formation and death *in vitro* and *in vivo*: a potential therapy agent for lung cancer. Cancer Gene Ther 17: 256–265. 19893593 10.1038/cgt.2009.74

[bib29] Ebert, O, Shinozaki, K, Kournioti, C, Park, MS, García-Sastre, A and Woo, SL (2004). Syncytia induction enhances the oncolytic potential of vesicular stomatitis virus in virotherapy for cancer. Cancer Res 64: 3265–3270. 15126368 10.1158/0008-5472.can-03-3753

[bib30] Nakamori, M, Fu, X, Meng, F, Jin, A, Tao, L, Bast, RC Jr et al. (2003). Effective therapy of metastatic ovarian cancer with an oncolytic herpes simplex virus incorporating two membrane fusion mechanisms. Clin Cancer Res 9: 2727–2733. 12855653

[bib31] Altomonte, J, Marozin, S, Schmid, RM and Ebert, O (2010). Engineered newcastle disease virus as an improved oncolytic agent against hepatocellular carcinoma. Mol Ther 18: 275–284. 19809404 10.1038/mt.2009.231 PMC2839313

[bib32] Gainey, MD, Manuse, MJ and Parks, GD (2008). A hyperfusogenic F protein enhances the oncolytic potency of a paramyxovirus simian virus 5 P/V mutant without compromising sensitivity to type I interferon. J Virol 82: 9369–9380. 18667520 10.1128/JVI.01054-08 PMC2546974

[bib33] Bateman, A, Bullough, F, Murphy, S, Emiliusen, L, Lavillette, D, Cosset, FL et al. (2000). Fusogenic membrane glycoproteins as a novel class of genes for the local and immune-mediated control of tumor growth. Cancer Res 60: 1492–1497. 10749110

[bib34] Galanis, E, Bateman, A, Johnson, K, Diaz, RM, James, CD, Vile, R et al. (2001). Use of viral fusogenic membrane glycoproteins as novel therapeutic transgenes in gliomas. Hum Gene Ther 12: 811–821. 11339897 10.1089/104303401750148766

[bib35] Allen, C, McDonald, C, Giannini, C, Peng, KW, Rosales, G, Russell, SJ et al. (2004). Adenoviral vectors expressing fusogenic membrane glycoproteins activated via matrix metalloproteinase cleavable linkers have significant antitumor potential in the gene therapy of gliomas. J Gene Med 6: 1216–1227. 15459967 10.1002/jgm.616

[bib36] Hoffmann, D, Bayer, W and Wildner, O (2007). In situ tumor vaccination with adenovirus vectors encoding measles virus fusogenic membrane proteins and cytokines. World J Gastroenterol 13: 3063–3070. 17589921 10.3748/wjg.v13.i22.3063 PMC4172612

[bib37] Hoffmann, D, Bayer, W and Wildner, O (2007). Local and distant immune-mediated control of colon cancer growth with fusogenic membrane glycoproteins in combination with viral oncolysis. Hum Gene Ther 18: 435–450. 17518612 10.1089/hum.2006.185

[bib38] Washburn, B and Schirrmacher, V (2002). Human tumor cell infection by Newcastle Disease Virus leads to upregulation of HLA and cell adhesion molecules and to induction of interferons, chemokines and finally apoptosis. Int J Oncol 21: 85–93. 12063554 10.3892/ijo.21.1.85

[bib39] Zamarin, D, Holmgaard, RB, Subudhi, SK, Park, JS, Mansour, M, Palese, P et al. (2014). Localized oncolytic virotherapy overcomes systemic tumor resistance to immune checkpoint blockade immunotherapy. Sci Transl Med 6: 226ra32. 10.1126/scitranslmed.3008095 PMC4106918 24598590

[bib40] Palucka, K and Banchereau, J (2012). Cancer immunotherapy via dendritic cells. Nat Rev Cancer 12: 265–277. 22437871 10.1038/nrc3258 PMC3433802

[bib41] Gallucci, S and Matzinger, P (2001). Danger signals: SOS to the immune system. Curr Opin Immunol 13: 114–119. 11154927 10.1016/s0952-7915(00)00191-6

[bib42] Kumar, H, Kawai, T and Akira, S (2011). Pathogen recognition by the innate immune system. Int Rev Immunol 30: 16–34. 21235323 10.3109/08830185.2010.529976

[bib43] Tang, D, Kang, R, Coyne, CB, Zeh, HJ and Lotze, MT (2012). PAMPs and DAMPs: signal 0s that spur autophagy and immunity. Immunol Rev 249: 158–175. 22889221 10.1111/j.1600-065X.2012.01146.x PMC3662247

[bib44] Guo, ZS, Liu, Z and Bartlett, DL (2014). Oncolytic immunotherapy: dying the right way is a key to eliciting potent antitumor immunity. Front Oncol 4: 74. 24782985 10.3389/fonc.2014.00074 PMC3989763

[bib45] Ostrand-Rosenberg, S, Sinha, P, Beury, DW and Clements, VK (2012). Cross-talk between myeloid-derived suppressor cells (MDSC), macrophages, and dendritic cells enhances tumor-induced immune suppression. Semin Cancer Biol 22: 275–281. 22313874 10.1016/j.semcancer.2012.01.011 PMC3701942

[bib46] Bartlett, DL, Liu, Z, Sathaiah, M, Ravindranathan, R, Guo, Z, He, Y et al. (2013). Oncolytic viruses as therapeutic cancer vaccines. Mol Cancer 12: 103. 24020520 10.1186/1476-4598-12-103 PMC3847443

[bib47] Inoue, H and Tani, K (2014). Multimodal immunogenic cancer cell death as a consequence of anticancer cytotoxic treatments. Cell Death Differ 21: 39–49. 23832118 10.1038/cdd.2013.84 PMC3857623

[bib48] Svet-Moldavsky, GJ and Hamburg, VP (1964). Quantitative relationships in viral oncolysis and the possibility of artificial heterogenization of tumours. Nature 202: 303–304. 10.1038/202303b0 14167800

[bib49] Kuzumaki, N, Moriuchi, T, Kodama, T and Kobayashi, H (1976). Xenogenization of rat erythroid cells by lymphatic leukemia virus: its role in induction of autoimmune hemolytic anemia. J Immunol 117: 1250–1255. 977949

[bib50] Donnelly, OG, Errington-Mais, F, Steele, L, Hadac, E, Jennings, V, Scott, K et al. (2013). Measles virus causes immunogenic cell death in human melanoma. Gene Ther 20: 7–15. 22170342 10.1038/gt.2011.205 PMC3378495

[bib51] Gauvrit, A, Brandler, S, Sapede-Peroz, C, Boisgerault, N, Tangy, F and Gregoire, M (2008). Measles virus induces oncolysis of mesothelioma cells and allows dendritic cells to cross-prime tumor-specific CD8 response. Cancer Res 68: 4882–4892. 18559536 10.1158/0008-5472.CAN-07-6265

[bib52] Guillerme, JB, Marc, G, Tangy, F, and Fonteneau, JF (2013). Antitumor virotherapy by attenuated measles virus. Biology 2: 587–602. 24832799 10.3390/biology2020587 PMC3960896

[bib53] Siddiqui, MA and Malathi, K (2012). RNase L induces autophagy via c-Jun N-terminal kinase and double-stranded RNA-dependent protein kinase signaling pathways. J Biol Chem 287: 43651–43664. 23109342 10.1074/jbc.M112.399964 PMC3527951

[bib54] Choi, AM, Ryter, SW and Levine, B (2013). Autophagy in human health and disease. N Engl J Med 368: 651–662. 23406030 10.1056/NEJMra1205406

[bib55] Levine, B and Deretic, V (2007). Unveiling the roles of autophagy in innate and adaptive immunity. Nat Rev Immunol 7: 767–777. 17767194 10.1038/nri2161 PMC7097190

[bib56] Richetta, C, Grégoire, IP, Verlhac, P, Azocar, O, Baguet, J, Flacher, M et al. (2013). Sustained autophagy contributes to measles virus infectivity. PLoS Pathog 9: e1003599. 24086130 10.1371/journal.ppat.1003599 PMC3784470

[bib57] Meng, C, Qiu, X, Jin, S, Yu, S, Chen, H and Ding, C (2012). Whole genome sequencing and biological characterization of Duck/JS/10, a new lentogenic class I Newcastle disease virus. Arch Virol 157: 869–880. 22310996 10.1007/s00705-012-1248-4

[bib58] Delpeut, S, Rudd, PA, Labonte, P, and von Messling, V (2012). Membrane fusion-mediated autophagy induction enhances morbillivirus cell-to-cell spread. J Virol 86: 8527–8535. 22647692 10.1128/JVI.00807-12 PMC3421762

[bib59] Joubert, PE, Meiffren, G, Grégoire, IP, Pontini, G, Richetta, C, Flacher, M et al. (2009). Autophagy induction by the pathogen receptor CD46. Cell Host Microbe 6: 354–366. 19837375 10.1016/j.chom.2009.09.006

[bib60] Park, MS, Shaw, ML, Muñoz-Jordan, J, Cros, JF, Nakaya, T, Bouvier, N et al. (2003). Newcastle disease virus (NDV)-based assay demonstrates interferon-antagonist activity for the NDV V protein and the Nipah virus V, W, and C proteins. J Virol 77: 1501–1511. 12502864 10.1128/JVI.77.2.1501-1511.2003 PMC140815

[bib61] Gotoh, B, Komatsu, T, Takeuchi, K and Yokoo, J (2001). Paramyxovirus accessory proteins as interferon antagonists. Microbiol Immunol 45: 787–800. 11838896 10.1111/j.1348-0421.2001.tb01315.x

[bib62] Childs, K, Randall, R and Goodbourn, S (2012). Paramyxovirus V proteins interact with the RNA Helicase LGP2 to inhibit RIG-I-dependent interferon induction. J Virol 86: 3411–3421. 22301134 10.1128/JVI.06405-11 PMC3302505

[bib63] Takeuchi, K, Kadota, SI, Takeda, M, Miyajima, N and Nagata, K (2003). Measles virus V protein blocks interferon (IFN)-alpha/beta but not IFN-gamma signaling by inhibiting STAT1 and STAT2 phosphorylation. FEBS Lett 545: 177–182. 12804771 10.1016/s0014-5793(03)00528-3

[bib64] Takayama, I, Sato, H, Watanabe, A, Omi-Furutani, M, Sugai, A, Kanki, K et al. (2012). The nucleocapsid protein of measles virus blocks host interferon response. Virology 424: 45–55. 22226324 10.1016/j.virol.2011.12.011

[bib65] Shaffer, JA, Bellini, WJ and Rota, PA (2003). The C protein of measles virus inhibits the type I interferon response. Virology 315: 389–397. 14585342 10.1016/s0042-6822(03)00537-3

[bib66] Zimmermann, M, Armeanu-Ebinger, S, Bossow, S, Lampe, J, Smirnow, I, Schenk, A et al. (2014). Attenuated and protease-profile modified sendai virus vectors as a new tool for virotherapy of solid tumors. PLoS ONE 9: e90508. 24598703 10.1371/journal.pone.0090508 PMC3944018

[bib67] Cantell, K, Hirvonen, S, Kauppinen, HL and Myllylä, G (1981). Production of interferon in human leukocytes from normal donors with the use of Sendai virus. Meth Enzymol 78(Pt A): 29–38. 6173603 10.1016/0076-6879(81)78094-7

[bib68] Hua, J, Liao, MJ and Rashidbaigi, A (1996). Cytokines induced by Sendai virus in human peripheral blood leukocytes. J Leukoc Biol 60: 125–128. 8699116 10.1002/jlb.60.1.125

[bib69] Nyman, TA, Tölö, H, Parkkinen, J and Kalkkinen, N (1998). Identification of nine interferon-alpha subtypes produced by Sendai virus-induced human peripheral blood leucocytes. Biochem J 329(Pt 2): 295–302. 9425112 10.1042/bj3290295 PMC1219044

[bib70] Costas, MA, Mella, D, Criscuolo, M, Díaz, A, Finkielman, S, Nahmod, VE et al. (1993). Superinduction of mitogen-stimulated interferon-gamma production and other lymphokines by Sendai virus. J Interferon Res 13: 407–412. 8151134 10.1089/jir.1993.13.407

[bib71] Zeng, J, Fournier, P and Schirrmacher, V (2002). Induction of interferon-alpha and tumor necrosis factor-related apoptosis-inducing ligand in human blood mononuclear cells by hemagglutinin-neuraminidase but not F protein of Newcastle disease virus. Virology 297: 19–30. 12083832 10.1006/viro.2002.1413

[bib72] Fournier, P, Zeng, J and Schirrmacher, V (2003). Two ways to induce innate immune responses in human PBMCs: paracrine stimulation of IFN-alpha responses by viral protein or dsRNA. Int J Oncol 23: 673–680. 12888903

[bib73] Schirrmacher, V and Fournier, P (2009). Newcastle disease virus: a promising vector for viral therapy, immune therapy, and gene therapy of cancer. Methods Mol Biol 542: 565–605. 19565923 10.1007/978-1-59745-561-9_30 PMC7122391

[bib74] Wang, BX, Rahbar, R and Fish, EN (2011). Interferon: current status and future prospects in cancer therapy. J Interferon Cytokine Res 31: 545–552. 21323567 10.1089/jir.2010.0158

[bib75] Williams, RF, Sims, TL, Tracey, L, Myers, AL, Ng, CY, Poppleton, H et al. (2010). Maturation of tumor vasculature by interferon-beta disrupts the vascular niche of glioma stem cells. Anticancer Res 30: 3301–3308. 20944101

[bib76] Borden, EC, Sen, GC, Uze, G, Silverman, RH, Ransohoff, RM, Foster, GR et al. (2007). Interferons at age 50: past, current and future impact on biomedicine. Nat Rev Drug Discov 6: 975–990. 18049472 10.1038/nrd2422 PMC7097588

[bib77] Zaidi, MR and Merlino, G (2011). The two faces of interferon-γ in cancer. Clin Cancer Res 17: 6118–6124. 21705455 10.1158/1078-0432.CCR-11-0482 PMC3186825

[bib78] Bekisz, J, Sato, Y, Johnson, C, Husain, SR, Puri, RK and Zoon, KC (2013). Immunomodulatory effects of interferons in malignancies. J Interferon Cytokine Res 33: 154–161. 23570381 10.1089/jir.2012.0167 PMC3647483

[bib79] Seliger, B, Ruiz-Cabello, F and Garrido, F (2008). IFN inducibility of major histocompatibility antigens in tumors. Adv Cancer Res 101: 249–276. 19055946 10.1016/S0065-230X(08)00407-7 PMC7125809

[bib80] Ikeda, H, Old, LJ and Schreiber, RD (2002). The roles of IFN gamma in protection against tumor development and cancer immunoediting. Cytokine Growth Factor Rev 13: 95–109. 11900986 10.1016/s1359-6101(01)00038-7

[bib81] Dunn, GP, Bruce, AT, Sheehan, KC, Shankaran, V, Uppaluri, R, Bui, JD et al. (2005). A critical function for type I interferons in cancer immunoediting. Nat Immunol 6: 722–729. 15951814 10.1038/ni1213

[bib82] Saga, K, Tamai, K, Yamazaki, T and Kaneda, Y (2013). Systemic administration of a novel immune-stimulatory pseudovirion suppresses lung metastatic melanoma by regionally enhancing IFN-γ production. Clin Cancer Res 19: 668–679. 23251005 10.1158/1078-0432.CCR-12-1947

[bib83] Kurooka, M and Kaneda, Y (2007). Inactivated Sendai virus particles eradicate tumors by inducing immune responses through blocking regulatory T cells. Cancer Res 67: 227–236. 17210703 10.1158/0008-5472.CAN-06-1615

[bib84] Suzuki, H, Kurooka, M, Hiroaki, Y, Fujiyoshi, Y and Kaneda, Y (2008). Sendai virus F glycoprotein induces IL-6 production in dendritic cells in a fusion-independent manner. FEBS Lett 582: 1325–1329. 18358837 10.1016/j.febslet.2008.03.011

[bib85] Fujihara, A, Kurooka, M, Miki, T and Kaneda, Y (2008). Intratumoral injection of inactivated Sendai virus particles elicits strong antitumor activity by enhancing local CXCL10 expression and systemic NK cell activation. Cancer Immunol Immunother 57: 73–84. 17602226 10.1007/s00262-007-0351-y PMC11030187

[bib86] Dufour, JH, Dziejman, M, Liu, MT, Leung, JH, Lane, TE and Luster, AD (2002). IFN-gamma-inducible protein 10 (IP-10; CXCL10)-deficient mice reveal a role for IP-10 in effector T cell generation and trafficking. J Immunol 168: 3195–3204. 11907072 10.4049/jimmunol.168.7.3195

[bib87] Balkwill, F (2004). Cancer and the chemokine network. Nat Rev Cancer 4: 540–550. 15229479 10.1038/nrc1388

[bib88] Lorence, RM, Rood, PA and Kelley, KW (1988). Newcastle disease virus as an antineoplastic agent: induction of tumor necrosis factor-alpha and augmentation of its cytotoxicity. J Natl Cancer Inst 80: 1305–1312. 2459402 10.1093/jnci/80.16.1305

[bib89] Zorn, U, Dallmann, I, Grosse, J, Kirchner, H, Poliwoda, H and Atzpodien, J (1994). Induction of cytokines and cytotoxicity against tumor cells by Newcastle disease virus. Cancer Biother 9: 225–235. 7820184 10.1089/cbr.1994.9.225

[bib90] Washburn, B, Weigand, MA, Grosse-Wilde, A, Janke, M, Stahl, H, Rieser, E et al. (2003). TNF-related apoptosis-inducing ligand mediates tumoricidal activity of human monocytes stimulated by Newcastle disease virus. J Immunol 170: 1814–1821. 12574346 10.4049/jimmunol.170.4.1814

[bib91] Schirrmacher, V, Bai, L, Umansky, V, Yu, L, Xing, Y and Qian, Z (2000). Newcastle disease virus activates macrophages for anti-tumor activity. Int J Oncol 16: 363–373. 10639582

[bib92] Vivier, E, Raulet, DH, Moretta, A, Caligiuri, MA, Zitvogel, L, Lanier, LL et al. (2011). Innate or adaptive immunity? The example of natural killer cells. Science 331: 44–49. 21212348 10.1126/science.1198687 PMC3089969

[bib93] Jarahian, M, Watzl, C, Fournier, P, Arnold, A, Djandji, D, Zahedi, S et al. (2009). Activation of natural killer cells by newcastle disease virus hemagglutinin-neuraminidase. J Virol 83: 8108–8121. 19515783 10.1128/JVI.00211-09 PMC2715740

[bib94] Arnon, TI, Lev, M, Katz, G, Chernobrov, Y, Porgador, A and Mandelboim, O (2001). Recognition of viral hemagglutinins by NKp44 but not by NKp30. Eur J Immunol 31: 2680–2689. 11536166 10.1002/1521-4141(200109)31:9<2680::aid-immu2680>3.0.co;2-a

[bib95] Mandelboim, O, Lieberman, N, Lev, M, Paul, L, Arnon, TI, Bushkin, Y et al. (2001). Recognition of haemagglutinins on virus-infected cells by NKp46 activates lysis by human NK cells. Nature 409: 1055–1060. 11234016 10.1038/35059110

[bib96] Arnon, TI, Achdout, H, Lieberman, N, Gazit, R, Gonen-Gross, T, Katz, G et al. (2004). The mechanisms controlling the recognition of tumor- and virus-infected cells by NKp46. Blood 103: 664–672. 14504081 10.1182/blood-2003-05-1716

[bib97] Chinnery, F, King, CA, Elliott, T, Bateman, AR and James, E (2012). Viral antigen mediated NKp46 activation of NK cells results in tumor rejection via NK-DC crosstalk. Oncoimmunology 1: 874–883. 23162755 10.4161/onci.20636 PMC3489743

[bib98] Harada, Y and Yonemitsu, Y (2011). Dramatic improvement of DC-based immunotherapy against various malignancies. Front Biosci (Landmark Ed) 16: 2233–2242. 21622173 10.2741/3850

[bib99] Okano, S, Yonemitsu, Y, Shirabe, K, Kakeji, Y, Maehara, Y, Harada, M et al. (2011). Provision of continuous maturation signaling to dendritic cells by RIG-I-stimulating cytosolic RNA synthesis of Sendai virus. J Immunol 186: 1828–1839. 21187441 10.4049/jimmunol.0901641

[bib100] Sugiyama, M, Kakeji, Y, Tsujitani, S, Harada, Y, Onimaru, M, Yoshida, K et al. (2011). Antagonism of VEGF by genetically engineered dendritic cells is essential to induce antitumor immunity against malignant ascites. Mol Cancer Ther 10: 540–549. 21209070 10.1158/1535-7163.MCT-10-0479

[bib101] Yoneyama, Y, Ueda, Y, Akutsu, Y, Matsunaga, A, Shimada, H, Kato, T et al. (2007). Development of immunostimulatory virotherapy using non-transmissible Sendai virus-activated dendritic cells. Biochem Biophys Res Commun 355: 129–135. 17292867 10.1016/j.bbrc.2007.01.132

[bib102] Yonemitsu, Y, Ueda, Y, Kinoh, H and Hasegawa, M (2008). Immunostimulatory virotherapy using recombinant Sendai virus as a new cancer therapeutic regimen. Front Biosci 13: 1892–1898. 17981677 10.2741/2809

[bib103] Komaru, A, Ueda, Y, Furuya, A, Tanaka, S, Yoshida, K, Kato, T et al. (2009). Sustained and NK/CD4+ T cell-dependent efficient prevention of lung metastasis induced by dendritic cells harboring recombinant Sendai virus. J Immunol 183: 4211–4219. 19734206 10.4049/jimmunol.0803845

[bib104] Kato, T, Ueda, Y, Kinoh, H, Yoneyama, Y, Matsunaga, A, Komaru, A et al. (2010). RIG-I helicase-independent pathway in sendai virus-activated dendritic cells is critical for preventing lung metastasis of AT6.3 prostate cancer. Neoplasia 12: 906–914. 21076616 10.1593/neo.10732 PMC2978913

[bib105] Schirrmacher, V, Haas, C, Bonifer, R and Ertel, C (1997). Virus potentiation of tumor vaccine T-cell stimulatory capacity requires cell surface binding but not infection. Clin Cancer Res 3: 1135–1148. 9815793

[bib106] Pearlstein, E, Salk, PL, Yogeeswaran, G and Karpatkin, S (1980). Correlation between spontaneous metastatic potential, platelet-aggregating activity of cell surface extracts, and cell surface sialylation in 10 metastatic-variant derivatives of a rat renal sarcoma cell line. Proc Natl Acad Sci USA 77: 4336–4339. 6933486 10.1073/pnas.77.7.4336 PMC349829

[bib107] Yogeeswaran, G and Salk, PL (1981). Metastatic potential is positively correlated with cell surface sialylation of cultured murine tumor cell lines. Science 212: 1514–1516. 7233237 10.1126/science.7233237

[bib108] Narayanan, S (1994). Sialic acid as a tumor marker. Ann Clin Lab Sci 24: 376–384. 7944275

[bib109] Komminoth, P, Roth, J, Saremaslani, P, Matias-Guiu, X, Wolfe, HJ and Heitz, PU (1994). Polysialic acid of the neural cell adhesion molecule in the human thyroid: a marker for medullary thyroid carcinoma and primary C-cell hyperplasia. An immunohistochemical study on 79 thyroid lesions. Am J Surg Pathol 18: 399–411. 8141431 10.1097/00000478-199404000-00008

[bib110] Sata, T, Roth, J, Zuber, C, Stamm, B and Heitz, PU (1991). Expression of alpha 2,6-linked sialic acid residues in neoplastic but not in normal human colonic mucosa. A lectin-gold cytochemical study with Sambucus nigra and Maackia amurensis lectins. Am J Pathol 139: 1435–1448. 1661075 PMC1886452

[bib111] Pousset, D, Piller, V, Bureaud, N, Monsigny, M and Piller, F (1997). Increased alpha2,6 sialylation of N-glycans in a transgenic mouse model of hepatocellular carcinoma. Cancer Res 57: 4249–4256. 9331085

[bib112] Fernández-Rodríguez, J, Feijoo-Carnero, C, Merino-Trigo, A, Páez de la Cadena, M, Rodríguez-Berrocal, FJ, de Carlos, A et al. (2000). Immunohistochemical analysis of sialic acid and fucose composition in human colorectal adenocarcinoma. Tumour Biol 21: 153–164. 10754466 10.1159/000030122

[bib113] Lu, DY, Lu, TR and Wu, HY (2012). Development of antimetastatic drugs by targeting tumor sialic acids. Sci Pharm 80: 497–508. 23008802 10.3797/scipharm.1205-01 PMC3447616

[bib114] Vierbuchen, M, Schröder, S, Larena, A, Uhlenbruck, G and Fischer, R (1994). Native and sialic acid masked Lewis(a) antigen reactivity in medullary thyroid carcinoma. Distinct tumour-associated and prognostic relevant antigens. Virchows Arch 424: 205–211. 8180782 10.1007/BF00193501

[bib115] Cohen, M, Elkabets, M, Perlmutter, M, Porgador, A, Voronov, E, Apte, RN et al. (2010). Sialylation of 3-methylcholanthrene-induced fibrosarcoma determines antitumor immune responses during immunoediting. J Immunol 185: 5869–5878. 20956342 10.4049/jimmunol.1001635

[bib116] Kingsbury, DW (1991). The Paramyxoviruses. Plenum Press: New York.

[bib117] Enders, G (1996). Paramyxoviruses. University of Texas Medical Branch at Galveston: Galveston. 21413341

[bib118] Powell, LD, Whiteheart, SW and Hart, GW (1987). Cell surface sialic acid influences tumor cell recognition in the mixed lymphocyte reaction. J Immunol 139: 262–270. 2953814

[bib119] Miest, TS and Cattaneo, R (2014). New viruses for cancer therapy: meeting clinical needs. Nat Rev Microbiol 12: 23–34. 24292552 10.1038/nrmicro3140 PMC4002503

[bib120] Pond, AR and Manuelidis, EE (1964). Oncolytic effect of poliomyelitis virus on human epidermoid carcinoma (hela tumor) heterologously transplanted to guinea pigs. Am J Pathol 45: 233–249. 14202523 PMC1907181

[bib121] Tsypkin, LB, Voroshilova, MK, Goryunova, AG, Lavrova, IK and Koroleva, GA (1976). The morphology of tumors of the human gastrointestinal tract in short-term organ culture and the reaction of these tumors to infection with poliovirus. Cancer 38: 1796–1806. 186175 10.1002/1097-0142(197610)38:4<1796::aid-cncr2820380457>3.0.co;2-y

[bib122] Toyoda, H, Wimmer, E and Cello, J (2011). Oncolytic poliovirus therapy and immunization with poliovirus-infected cell lysate induces potent antitumor immunity against neuroblastoma in vivo. Int J Oncol 38: 81–87. 21109928

[bib123] Chumakov, PM, Morozova, VV, Babkin, IV, Baikov, IK, Netesov, SV and Tikunova, NV (2012). [Oncolytic enteroviruses]. Mol Biol (Mosk) 46: 712–725. 23156670

[bib124] Twigger, K, Roulstone, V, Kyula, J, Karapanagiotou, EM, Syrigos, KN, Morgan, R et al. (2012). Reovirus exerts potent oncolytic effects in head and neck cancer cell lines that are independent of signalling in the EGFR pathway. BMC Cancer 12: 368. 22920673 10.1186/1471-2407-12-368 PMC3537694

[bib125] Rommelaere, J, Geletneky, K, Angelova, AL, Daeffler, L, Dinsart, C, Kiprianova, I et al. (2010). Oncolytic parvoviruses as cancer therapeutics. Cytokine Growth Factor Rev 21: 185–195. 20211577 10.1016/j.cytogfr.2010.02.011

[bib126] Kirn, DH and McCormick, F (1996). Replicating viruses as selective cancer therapeutics. Mol Med Today 2: 519–527. 9015793 10.1016/s1357-4310(97)81456-6

[bib127] Sviatchenko, VA, Tarasova, MV, Netesov, SV and Chumakov, PM (2012). [Oncolytic adenoviruses in anti-cancer therapy: current status and perspectives]. Mol Biol (Mosk) 46: 556–569. 23113343

[bib128] Senzer, NN, Kaufman, HL, Amatruda, T, Nemunaitis, M, Reid, T, Daniels, G et al. (2009). Phase II clinical trial of a granulocyte-macrophage colony-stimulating factor-encoding, second-generation oncolytic herpesvirus in patients with unresectable metastatic melanoma. J Clin Oncol 27: 5763–5771. 19884534 10.1200/JCO.2009.24.3675

[bib129] Barber, GN (2004). Vesicular stomatitis virus as an oncolytic vector. Viral Immunol 17: 516–527. 15671748 10.1089/vim.2004.17.516

[bib130] Nakamura, T and Russell, SJ (2004). Oncolytic measles viruses for cancer therapy. Expert Opin Biol Ther 4: 1685–1692. 15461580 10.1517/14712598.4.10.1685

[bib131] Kirn, D, and Caroline, B (2008). Oncolytic vaccinia virus cancer therapy. US patents 20100303714 A1 and 8747837 B2.

[bib132] Kochneva, GV, Sivolobova, GF, Yudina, KV, Babkin, IV, Chumakov, PM, and Netesov, SV (2012). Oncolytic poxviruses. Mol Genet Microbiol Virol 27: 8–15. 22702138

[bib133] Bian, H, Fournier, P, Moormann, R, Peeters, B and Schirrmacher, V (2005). Selective gene transfer to tumor cells by recombinant Newcastle Disease Virus via a bispecific fusion protein. Int J Oncol 26: 431–439. 15645128

[bib134] Msaouel, P, Iankov, ID, Allen, C, Russell, SJ and Galanis, E (2012). Oncolytic measles virus retargeting by ligand display. Methods Mol Biol 797: 141–162. 21948475 10.1007/978-1-61779-340-0_11 PMC3691680

[bib135] Nussbaum, O, Broder, CC, Moss, B, Stern, LB, Rozenblatt, S and Berger, EA (1995). Functional and structural interactions between measles virus hemagglutinin and CD46. J Virol 69: 3341–3349. 7745681 10.1128/jvi.69.6.3341-3349.1995 PMC189046

[bib136] Allen, C, Vongpunsawad, S, Nakamura, T, James, CD, Schroeder, M, Cattaneo, R et al. (2006). Retargeted oncolytic measles strains entering via the EGFRvIII receptor maintain significant antitumor activity against gliomas with increased tumor specificity. Cancer Res 66: 11840–11850. 17178881 10.1158/0008-5472.CAN-06-1200

[bib137] Nakamura, T, Peng, KW, Harvey, M, Greiner, S, Lorimer, IA, James, CD et al. (2005). Rescue and propagation of fully retargeted oncolytic measles viruses. Nat Biotechnol 23: 209–214. 15685166 10.1038/nbt1060

[bib138] Paraskevakou, G, Allen, C, Nakamura, T, Zollman, P, James, CD, Peng, KW et al. (2007). Epidermal growth factor receptor (EGFR)-retargeted measles virus strains effectively target EGFR- or EGFRvIII expressing gliomas. Mol Ther 15: 677–686. 17299404 10.1038/sj.mt.6300105

[bib139] Hasegawa, K, Hu, C, Nakamura, T, Marks, JD, Russell, SJ and Peng, KW (2007). Affinity thresholds for membrane fusion triggering by viral glycoproteins. J Virol 81: 13149–13157. 17804513 10.1128/JVI.01415-07 PMC2169077

[bib140] Hasegawa, K, Nakamura, T, Harvey, M, Ikeda, Y, Oberg, A, Figini, M et al. (2006). The use of a tropism-modified measles virus in folate receptor-targeted virotherapy of ovarian cancer. Clin Cancer Res 12(20 Pt 1): 6170–6178. 17062694 10.1158/1078-0432.CCR-06-0992

[bib141] Ungerechts, G, Springfeld, C, Frenzke, ME, Lampe, J, Johnston, PB, Parker, WB et al. (2007). Lymphoma chemovirotherapy: CD20-targeted and convertase-armed measles virus can synergize with fludarabine. Cancer Res 67: 10939–10947. 18006839 10.1158/0008-5472.CAN-07-1252

[bib142] Hallak, LK, Merchan, JR, Storgard, CM, Loftus, JC and Russell, SJ (2005). Targeted measles virus vector displaying echistatin infects endothelial cells via alpha(v)beta3 and leads to tumor regression. Cancer Res 65: 5292–5300. 15958576 10.1158/0008-5472.CAN-04-2879

[bib143] Allen, C, Paraskevakou, G, Iankov, I, Giannini, C, Schroeder, M, Sarkaria, J et al. (2008). Interleukin-13 displaying retargeted oncolytic measles virus strains have significant activity against gliomas with improved specificity. Mol Ther 16: 1556–1564. 18665158 10.1038/mt.2008.152 PMC2748750

[bib144] Peeters, BP, de Leeuw, OS, Koch, G and Gielkens, AL (1999). Rescue of Newcastle disease virus from cloned cDNA: evidence that cleavability of the fusion protein is a major determinant for virulence. J Virol 73: 5001–5009. 10233962 10.1128/jvi.73.6.5001-5009.1999 PMC112544

[bib145] Dortmans, JC, Koch, G, Rottier, PJ and Peeters, BP (2011). Virulence of Newcastle disease virus: what is known so far? Vet Res 42: 122. 22195547 10.1186/1297-9716-42-122 PMC3269386

[bib146] Zamarin, D and Palese, P (2012). Oncolytic Newcastle disease virus for cancer therapy: old challenges and new directions. Future Microbiol 7: 347–367. 22393889 10.2217/fmb.12.4 PMC4241685

[bib147] Vigil, A, Park, MS, Martinez, O, Chua, MA, Xiao, S, Cros, JF et al. (2007). Use of reverse genetics to enhance the oncolytic properties of Newcastle disease virus. Cancer Res 67: 8285–8292. 17804743 10.1158/0008-5472.CAN-07-1025

[bib148] Zamarin, D, Martínez-Sobrido, L, Kelly, K, Mansour, M, Sheng, G, Vigil, A et al. (2009). Enhancement of oncolytic properties of recombinant newcastle disease virus through antagonism of cellular innate immune responses. Mol Ther 17: 697–706. 19209145 10.1038/mt.2008.286 PMC2835121

[bib149] Zamarin, D, Vigil, A, Kelly, K, García-Sastre, A and Fong, Y (2009). Genetically engineered Newcastle disease virus for malignant melanoma therapy. Gene Ther 16: 796–804. 19242529 10.1038/gt.2009.14 PMC2882235

[bib150] Song, KY, Wong, J, Gonzalez, L, Sheng, G, Zamarin, D and Fong, Y (2010). Antitumor efficacy of viral therapy using genetically engineered Newcastle disease virus [NDV(F3aa)-GFP] for peritoneally disseminated gastric cancer. J Mol Med 88: 589–596. 20393691 10.1007/s00109-010-0605-6 PMC3269811

[bib151] Silberhumer, GR, Brader, P, Wong, J, Serganova, IS, Gönen, M, Gonzalez, SJ et al. (2010). Genetically engineered oncolytic Newcastle disease virus effectively induces sustained remission of malignant pleural mesothelioma. Mol Cancer Ther 9: 2761–2769. 20858727 10.1158/1535-7163.MCT-10-0090 PMC3266818

[bib152] Li, P, Chen, CH, Li, S, Givi, B, Yu, Z, Zamarin, D et al. (2011). Therapeutic effects of a fusogenic newcastle disease virus in treating head and neck cancer. Head Neck 33: 1394–1399. 21928411 10.1002/hed.21609 PMC3116983

[bib153] Elankumaran, S, Rockemann, D and Samal, SK (2006). Newcastle disease virus exerts oncolysis by both intrinsic and extrinsic caspase-dependent pathways of cell death. J Virol 80: 7522–7534. 16840332 10.1128/JVI.00241-06 PMC1563725

[bib154] Kinoh, H and Inoue, M (2008). New cancer therapy using genetically-engineered oncolytic Sendai virus vector. Front Biosci 13: 2327–2334. 17981715 10.2741/2847

[bib155] Kinoh, H, Inoue, M, Komaru, A, Ueda, Y, Hasegawa, M and Yonemitsu, Y (2009). Generation of optimized and urokinase-targeted oncolytic Sendai virus vectors applicable for various human malignancies. Gene Ther 16: 392–403. 19037241 10.1038/gt.2008.167

[bib156] Kantoff, PW, Higano, CS, Shore, ND, Berger, ER, Small, EJ, Penson, DF et al. IMPACT Study Investigators. (2010). Sipuleucel-T immunotherapy for castration-resistant prostate cancer. N Engl J Med 363: 411–422. 20818862 10.1056/NEJMoa1001294

[bib157] Surowiak, P, Materna, V, Maciejczyk, A, Kaplenko, I, Spaczynski, M, Dietel, M et al. (2006). CD46 expression is indicative of shorter revival-free survival for ovarian cancer patients. Anticancer Res 26(6C): 4943–4948. 17214367

[bib158] Maciejczyk, A, Szelachowska, J, Szynglarewicz, B, Szulc, R, Szulc, A, Wysocka, T et al. (2011). CD46 Expression is an unfavorable prognostic factor in breast cancer cases. Appl Immunohistochem Mol Morphol 19: 540–546. 21617523 10.1097/PAI.0b013e31821a0be9

[bib159] Noyce, RS, and Richardson, CD (2012). Nectin 4 is the epithelial cell receptor for measles virus. Trends Microbiol 19: 19. 10.1016/j.tim.2012.05.006 22721863

[bib160] Fabre-Lafay, S, Garrido-Urbani, S, Reymond, N, Gonçalves, A, Dubreuil, P and Lopez, M (2005). Nectin-4, a new serological breast cancer marker, is a substrate for tumor necrosis factor-alpha-converting enzyme (TACE)/ADAM-17. J Biol Chem 280: 19543–19550. 15784625 10.1074/jbc.M410943200

